# Effect of Surface Treatments on Adhesion of Milled Polyetheretherketone Post to Different Composite Resin Core Build-Up Materials

**DOI:** 10.4317/jced.60740

**Published:** 2023-09-01

**Authors:** Tarek-Ahmed Soliman, Eman-Mohamed Raffat-Hussein, Mohamed Ellayeh, Dina-Samy Farahat

**Affiliations:** 1 Dental Biomaterials Department, Faculty of Dentistry, Mansoura University, Mansoura, Egypt; 2 Removable prosthodontic Department, Misr International University, Cairo, Egypt; 3 Prosthetic Dentistry Department, General Zagazig Hospital, Egypt; 4 Fixed Prosthodontics Department, Faculty of Dentistry, Mansoura University, Mansoura, Egypte; 5 Prosthetic Dentistry Department, Faculty of Dentistry, New Mansoura University, New Mansoura, Egypt

## Abstract

**Background:**

To evaluate the effect of surface treatments on adhesion of milled PEEK post to two different composite resin core-build up materials.

**Material and Methods:**

Six PEEK posts were divided into the following groups: G1: no treatment+ Grandio core material, G2: no treatment+ Bulk-fill core material, G3: 98% sulfuric acid for 60 seconds+ Grandio core material, G4: 98% sulfuric acid for 60 seconds+ Bulk-fill core material, G5: 50 μm airborne particle abraded + Grandio core material, G6: 50 μm airborne particle abraded + bulk fill core material. The adhesion of the post to core build-up materials was tested using micro push-out bond strength. Data were analyzed using ANOVA and Tukey’s test.

**Results:**

There was a significant effect for the surface treatment (*p*<.001), a non-significant effect for the core build-up materials (*p*<.289), and a significant effect for their interaction (*p*<.001) on the bond strength values.

**Conclusions:**

Within the limitation of this study, sulfuric acid etching group significantly increased the bond strength compared to other groups. Bulk-fill core material could be a feasible option when restoring ETT in terms of saving chair time and the treatment procedure simplicity.

** Key words:**Bond Strength, Core material, PEEK, Surface Treatment.

## Introduction

Endodontically treated teeth (ETT) that have lost more than 50% of their coronal structure are vulnerable to shearing chewing forces and frequently require the use of a post-and-core. The final restoration is made more sTable and retained by using posts to hold the core material in place (1-3). For a composite resin core restoration to be durable and therefore successful clinically, there must be an appropriate bond between the resin core material and the post material (3-6). The surface treatment of the post (5,7), the post, and the composite resin core material (8) all have an impact on how well the composite core is kept attached to the prefabricated post.

The use of aesthetic prefabricated fiber posts is required by the increasing patient demand for aesthetic improvement (9-11). Because of their adequate aesthetics, uniform distribution of stress, biocompatibility, easy and rapid handling, and adhesion to resin materials, glass fiber posts is preferred over the metal posts for the rehabilitation of endodontically treated teeth (11-13). Fiber posts, on the other hand, can cause mechanical stress in the restoration margin and do not strengthen the tooth structure (14). Furthermore, despite having a lower elasticity modulus (45.7-53.8 GPa) than metal posts (95.0-110.0 GPa) (12,13), it is nearly three times greater than dentin (18.6 GPa). The failure of fiber post and core build up materials is caused by a variety of mechanisms, including resin matrix cracking, fiber fracture, and interfacial detachment (10,11). As a result, a low young modulus material with acceptable aesthetics has been developed (15).

PEEK is a thermoplastic polymer with excellent properties (16-19). Because it has a lower young modulus (3-4 GPa) than dentin, it can minimize stresses transferred to the restoration and teeth (20).Due to its intriguing properties, PEEK has been proposed for use in a variety of dental applications, including fixed prostheses, removable prostheses, custom post-and-core, endo crowns, interim restoration, dental implants, individual abutments, and orthodontic wires (21-23). However, bonding PEEK to resin materials remains challenging due to its resistance to surface alteration and low surface energy (24,25). Surface treatments with chemical and micromechanical retention have been suggested to enhance the composite resin core bonding to PEEK post (24,25).

The material used in the core build-up also influenced the interfacial bond strength. Composite resin is the material of choice for core build-up to reinforce missing tooth structure. However, it must be restored incrementally to guarantee proper polymerization. Multiple increments may leave voids and gaps due to the difficulty of placement in a deep cavity. As a result, bulk fill resin composites were developed to address the issue of layering techniques while also saving time during the restorative procedure (26). PEEK may possibly be a viable alternative for restoration of ETT (27,28). Nevertheless, little research has been conducted for the evaluation of PEEK as a post material with efficient surface treatments and composite resin-core build up materials. As a result, the purpose of this research was to evaluate the effect of surface treatment on adhesion of milled PEEK post to two different types of composite resin core-build up materials. The null hypotheses proposed that 1) core-build up materials, 2) the surface treatments conditions and 3) their interactions would have no effect on the bond strength of milled PEEK post.

## Material and Methods

-Study materials

In this study, one type of post milled from a prefabricated PEEK blank (PK, Bredent GmbH &co., Senden, Germany) by using a CAD-CAM system, and two types of resin composites core build-up materials were used: incrementally added resin composite (Grandio core DC; GR, VOCO GmbH, Cuxhaven, Germany) and a high viscosity bulk-fill resin composite (X-tra fil; XF, VOCO GmbH, Cuxhaven, Germany). The manufacturers and the compositions of the materials used in this study are presented in Table 1.

**Table 1:**
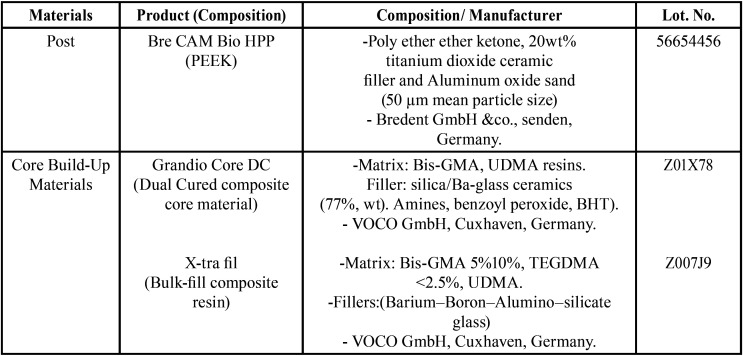
Materials used in the study.

-Specimen preparation and grouping

Six PEEK posts were machined from a prefabricated PEEK blank using a CAD-CAM system. Using an intraoral scanner (CEREC Prime scan; DENTSPLY Sirona), a fiber glass reinforced composite post (size #3) with a maximum diameter of 2 mm was digitally scanned to produce a standard tessellation language (STL) file. The post diameter and length were set at 2 mm and 12 mm, respectively, for standardization. The collected data were fed into a milling machine (CEREC inLab MC X5; Dentsply Sirona), which produced PEEK posts from a milling blank (Ø 98.5 mm, T12 mm).

The posts were divided into six groups (n = 1 post, 7 disc/group) according to the surface treatment method and the core build-up materials, as follows: Group 1(NTGR): no surface treatment was performed, and core build-up material was introduced incrementally (GR) (negative control group), Group 2(NTXF): no surface treatment was performed, and bulk-fill core-build-up material was used (XF) (positive control group), Group 3 (AEGR): 98% sulfuric acid for 60 seconds, rinsed with deionized water for 60 seconds, and core build-up material was introduced incrementally (GR), Group 4 (AEXF): the same procedure as group 3 and bulk-fill core-build-up material was applied (XF), Group5 (AAGR): airborne particle abraded with 50 μm aluminum oxide (LEMAT NT4, Wassermann, Germany) for 10 seconds at a distance of 10 mm with a pressure of 0.55 MPa, and then air-dried for 20 seconds and core build-up material was introduced incrementally (GR), Group 6 (AAXF):the same procedure as group 5 and bulk-fill core-build-up material was applied (XF). A Visio. Link primer (Bredent GmbH & Co., Senden, Germany) was applied on the surface of the post with a micro brush, gently air-dried for 60 s, and then immediately polymerized for 20 sec using a LED light (Elipar Freeligh 2, 3M ESPE, 1,226 mW/cm²), according to the manufacturer’s instruction.

-Core build-up procedure and micro push-out bond strength test

Core build-up was performed *in vitro* using a dual cure composite core material (GR) and a bulk-fill composite resin material (XF), as previously described by Goracci *et al*. (29). Each post was placed perpendicularly on a glass slab, and it was fastened with a drop of sticky wax. The post was then surrounded by a cylindrical plastic matrix with a diameter of 9.2 mm, and the core was constructed using the incremental technique (2-mm) for (GR group) and bulk fill technique (4-mm) for (XF group). The composite core build-up materials were cured for 20 seconds using Elipar Free Light 2 (3M; St Paul, MN, USA; light output: 1226 mW/cm2) from the matrix’s open upper side and through the post. After being filled with polymerized composite, the matrix was removed. As a result, a cylinder of composite resin was formed around the PEEK post. To ensure optimal polymerization of the resin composite material, the bottom side of the cylinder was light cured for an additional 20 seconds.

After the core build-up procedure was completed, the specimens were sectioned and loaded to simulate the clinical condition of immediate loading. Each bonded specimen was sectioned at low speed under cooling water with a diamond saw (Isomet 1000, Beuhler Ltd., Lake Bluff, IL, USA), yielding 7-disc specimens, each 1 mm thick. A digital caliper was used to measure the thickness of each disc (Mitutoyo, Tokyo, Japan). At a crosshead speed of 0.05 mm/min, each disc-specimen was loaded into a universal testing machine (Model TT-B, Instron Corp., Canton, MA, USA) with a 1 mm diameter cylindrical plunger centered on the disc and avoiding contact with the surrounding core surface. The micro push-out bond strength (MPa) was calculated by dividing the load at failure (Newtons) by the bonding area (mm2). The total bonding area for each post was determined using the formula (7): A=2r×π×h where r is the post radius (2 mm), π is the constant 3.14 and h is the thickness of each post section. Failure modes were classified into three categories using a stereomicroscope (Olympus SZX-ILLB100-Olympus Optical Co. Ltd., Tokyo, Japan) at 40-x magnification: Adhesive failure between the post and the core materials; cohesive failure within the post or the core material, and mixed failure. The following formula was used to calculate the percentage of failure modes for each group: Failure modes (%) = Nf/ Nt 100%, where Nf is the total number of specimens in each group and Nt is the number of specimens presented for each mode of failure.

-Scanning Electron Microscopy

A scanning electron microscope (SEM) (JEOL, JSM, 6510, Tokyo, Japan) was used to examine the morphological aspects of posts after surface treatments on an additional PEEK specimen from each group. The specimens were ultrasonically cleansed for 3 minutes with deionized water, then immersed in 96% ethanol for 2 minutes and air dried. Each specimen was sputter-coated with gold (Sputter Coater S150A; Japan) and examined under a microscope at 200 x magnification.

-Statistical analysis

According to the Shapiro-Wilk and Levene tests, the normality and equal variance assumptions were met. Following that, statistical analyses (SPSS 22.0; IBM statistics) of the micro-push out bond strength were performed using one-way ANOVA to determine statistically significant differences in the six groups. For post-hoc comparisons, Tukey’s significant difference test was used. To detect the interaction between the two independent variables (surface treatment and core build-up materials), a two-way ANOVA was also used. The Chi-square (χ2) was used to determine significant differences in the mode of failure analysis. The level of significance for all statistical tests was set at 5%.

## Results

Table 2 shows the means and standard deviations of micro push-out bond strength for all groups. The two-way ANOVA revealed a significant effect for the surface treatment (F= 102.989, *p* <.001), non-significant effect for the core build-up materials (F= 1.154, *p* <.289), and a significant effect for their interaction (F= 28.188, *p* <.001) on the bond strength values. One-way ANOVA indicated a statistically significant difference in all the six groups (F= 52.701, *p* <.001).

**Table 2:**
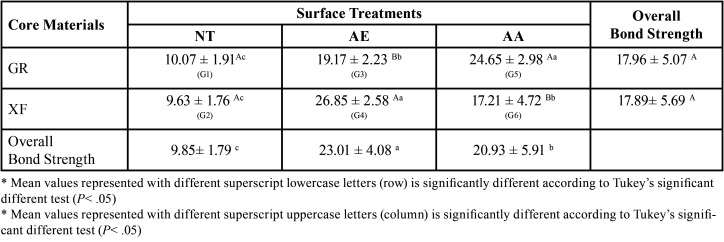
Micro Push-out Bond Strength for the Different Groups.

 There was a significant difference (*p* <.001) between G1 (NTGR, 10.07 ± 1.91) and all groups except G2 (NTXF, 9.63 ± 1.76) (*p* = 1). The results of the bond strength values achieved for AE (G3 and G4) and AA (G5 and G6) were significantly higher (*p* <.001) compared with the control groups for both types of the core build-up materials. Although the overall effect of the core build-up material showed no significant effect on the bond strength values, bulk fill core build-up material in G4 showed a higher significant effect (*p* <.001) on bond strength in comparison to incrementally added composite core build-up in G3 regarding sulfuric acid etching. Also, the incrementally added core material in G5 showed a higher significant effect on bond strength in comparison to the bulk fill one in G6 regarding the air born particle abrasion. The overall impact of surface treatment showed statistically significant effect (*p* <.05) on the bond strength values, with higher values for sulfuric acid etching group (23.01 ± 4.08) compared to air born particle abrasion (20.93 ± 5.91). The integrated effect of the surface treatment and the core build-up material showed a significant effect on the bond strength (*p* <.001). G 4 (AEXF) and G 5 (AAGR) showed the highest bond strength values among all tested groups (26.85 ± 2.58) and (24.65 ± 2.98) respectively. Furthermore, there was no significant difference between G 4 (AEXF) and G 5 (AAGR). In addition, there was no significant difference between G 6 (AAXF) and G 3 (AEGR) (*p* = .41).

**Figure 1:**
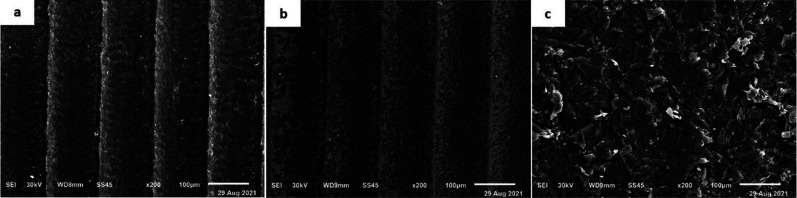
SEM micrographs (200 X) of PEEK post (a) No treatment group (b) Sulfuric acid etching group and (c) Air particle abrasion group.

For the failure mode, a predominance of adhesive failure between the PEEK post and the core build-up material was found in the no treatment group, while mixed failure was predominantly found in the AE and AA groups (Table 3). SEM images at 200× magnification is shown in Fig. 1a-c. The control group had a polished PEEK surface that was plain, smooth, and homogeneous (Fig. 1a). The specimen treated with 98% sulfuric acid was characterized by irregular surface of filler particles and small pits simulating a sponge-like network (Fig. 1b). The surface of the air born abrasion particle group was irregular and fissured, with polygonal-shaped alumina oxide embedded in it (Fig. 1c).

**Table 3:**
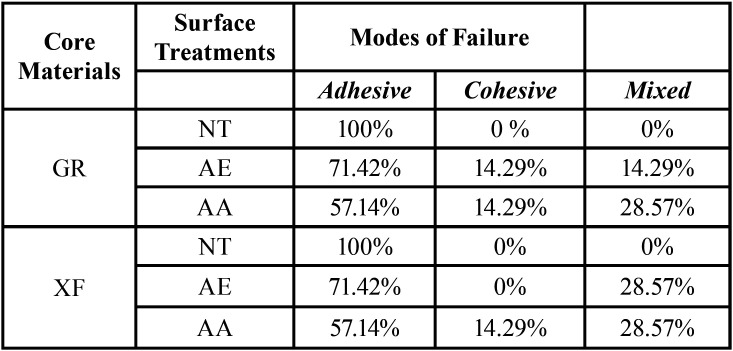
Modes of Failure.

## Discussion

PEEK post could be a viable option for the restoration of endodontically treated teeth especially when considering the cost-effectiveness. Furthermore, it has a modulus of elasticity similar to that of dentin, as well as high fracture resistance and biocompatibility (23,30). The effect of different surface treatments on the micro-push-out adhesion of PEEK posts to two different core build-up materials was investigated in this study. The results showed a significant effect of surface treatment, a non-significant effect of the core build-up materials and a significant effect of their interactions on the bond strength values. Thus, the first and third null hypotheses were rejected, but the second was accepted.

The micro-push-out test has been used to assess the adhesion of PEEK posts to resin core materials because it is more efficient, clinically relevant and provides a more accurate assessment of bond strength than the conventional shear test, as the fracture occurs parallel to the bonding interface rather than in the transverse direction, simulating clinical conditions (5,31). In addition, the ability to obtain multiple specimens from a single bonded post/composite core (1).

Various surface treatments have been proposed to improve the adhesion of composite resin cores to posts. Sulfuric acid etching is technique used to improve the surface properties of PEEK for bonding (24,25). The current study showed that 98% sulfuric acid etching of PEEK post recorded the highest overall bond strength values when compared to other groups. This could be attributed to the sulfonate groups produced by sulfuric acid, which are then chemically cross-linked to the dental adhesives (32,33). In addition, compared to the control group, SEM images showed that 98% sulfuric acid group changed from a plain homogeneous pattern to a blister-like surface with porosities that improve resin adhesive penetration, resulting in increased bond strength (Fig. 1b). The findings of this study are consistent with previous studies, which found that sulfuric acid etching had higher bond strength than hydrofluoric acid (25), argon plasma (25), air abrasion (50-110 m), and silica coating (17,25,34,35).

Airborne abrasion is a popular method of surface treatment in the field of dentistry. The bond strength value for PEEK post 50-µm airborne-particle abraded was significantly higher in comparison to the control group. This could be attributed to the increases in surface roughness, creates a fresh surface layer and promotes micromechanical interlocking with dental adhesives(25,27,28). In addition, SEM image (Fig. 1c) revealed an irregular fissure pattern with grooves and cracks, which could explain the improved bond strength over the control group.

The core-build up materials should exhibit good adaptation and a strong bond to the post surface. The micromechanical and chemical interaction between posts and composite resins could explain the retention of the core portion around the post (1). One of the factors that affects the adhesion of the post to the core material and the procedure’s simplicity is the thickness of the incremental layer. Bulk-fill composite resins were evaluated as a material for core build-up in this study because they make it easier to fill cavities to a depth of about 4 mm and rebuild structural loss in single incremental layers (36,37). The impact of composite polymerization techniques with universal adhesive on the push-out bond strength in coronal dentin was assessed by Moosavi *et al*. (38). When using universal adhesive in self-etch mode, they discovered that bulk-fill composites had greater bond strength values (15.36 ±5.17) than dual-cured composite resins (5.10 ±2.74). In endodontically treated teeth, Martins *et al*. (39) investigated the bond strength of glass fiber posts cemented with flowable bulk resin or resin cement. The results of this study showed that the bond strength of flowable bulk-fill was comparable to resin cement indicating that it is a possible alternative material for cementation.

Although the overall effect of the core build-up material had no significant effect on the bond strength values in this study, bulk fill had a lower significant effect (*p* <.001) on bond strength in comparison to the sulfuric acid etching group. This might be explained by its high viscosity, which prevents it from penetrating into the surface grooves. Furthermore, the incorporation of alumina powder in the PEEK surface may hinder adhesive penetration (Fig. 1c). However, compared to the airborne abrasion group, the bulk-fill demonstrated a significantly (*p* <.001) higher bond strength value in sulfuric acid etching group. This difference could be attributed to two factors: first, the sulfuric acid produces sulfonate groups in PEEK polymer chains, which are then chemically cross-linked to the dental adhesives (32,33), and second, the translucency of bulk fill, which makes it easier to achieve a greater depth of cure and hence the bond strength (40). Furthermore, the close matching of the elastic modulus of bulk fill composite resin (11.6 GPa) to PEEK material (18.6 GPa) is one of the factors that could affect its bond strength because it uniformly distributes stresses and reduces their transmission to the restorations, thereby improving its adaptation (18,27).

Although there is limited information on the bond strength of PEEK posts to bulk-fill core material, the results of this *in vitro* study show accepTable bond strength. Another issue that needs to be considered is the lack of information on the long-term performance of the PEEK posts. One limitation of this study was that the effect of oral environment was not considered. Further *in vitro* and *in vivo* research is needed to validate the current results and determine the durability of PEEK posts with an appropriate core build-up material.

## Conclusions

Based on the results presented, the following conclusions can be made:

1. The Sulfuric acid etching group significantly increased the bond strength compared to other groups. Another method for enhancing the bond strength of PEEK posts could be the airborne particle abrasion.

2. The overall effect of the core build-up material had no significant effect on PEEK post bond strength values. Thus bulk-fill core build-up material could be a viable option when restoring ETT in terms of saving chair time and the treatment procedure simplicity.

3. The combining effect of the PEEK post-surface treatment and the core-build up material significantly affects the interfacial bond strength.
